# Corrosion Behavior of GH4169 Alloy under Alternating Oxidation at 900 °C and Solution Immersion

**DOI:** 10.3390/ma12091503

**Published:** 2019-05-08

**Authors:** Zhongfen Yu, Li Liu, Rui Liu, Min Cao, Lei Fan, Ying Li, Shujiang Geng, Fuhui Wang

**Affiliations:** 1 School of metallurgy, Northeastern University, Shenyang, 110819, China; zfyu12s@imr.ac.cn (Z.Y.); gengshujiang@mail.neu.edu.cn (S.G.); 2 Institute of Metal Research, Chinese Academy of Sciences, Shenyang 110016, China; rliu15s@imr.ac.cn (R.L.); mcao12s@imr.ac.cn (M.C.); fanlei1@cnpc.com.cn (L.F.); liying@imr.ac.cn (Y.L.); 3 Key Laboratory for Anisotropy and Texture of Materials (MoE), School of Materials Science and Engineering, Northeastern University, Shenyang 110819, China; wangfh@imr.ac.cn

**Keywords:** Ni-based superalloy, high temperature oxidation

## Abstract

In this paper, the corrosion behavior of GH4169 superalloy under alternating oxidation (at 900 °C) and solution immersion (in 3.5% NaCl solution, 30 ± 1 °C) has been studied by SEM, XRD, XPS, and electron probe microanalysis (EPMA). The results show that the alternating environment increases the corrosion rate of GH4169. The reaction of NaCl and Cr_2_O_3_ generates various volatile and soluble corrosion products, such as Na_2_Cr_2_O_7_, CrCl_3_, Cl_2_, and Na_2_CrO_4_, at a high temperature. The destruction of the protective Cr_2_O_3_ film leads to the increase of defects in the oxide scale, promoting the formation of oxides, such as NiO and Fe_2_O_3_, and changes the composition and structure of the oxide film. After repeated iterations, the mixed oxides will result in the spalling of the oxide film because they can reduce the fracture toughness of the corrosion scale. Therefore, the corrosion is comprehensively intensified.

## 1. Introduction

Ni-based superalloys are widely used in the turbine blades of gas turbines and aircraft engines due to their excellent mechanical strength and oxidation resistance [1,2,3]. These materials, however, suffer serious corrosion in filed experiences [4,5,6]. Many researchers have focused on the high temperature oxidation behavior of Ni-based superalloy in pure O_2_ or air [7,8,9,10]. Previous investigations showed that the GH202 superalloy had excellent oxidation resistance at 800 °C and 900 °C due to the formation of a continuous and dense Cr_2_O_3_ layer, which improved the bonding strength between the oxide scale and the substrate [11]. Some scholars believe that the spalling of NiCr_2_O_4_ spinel could lead to the depletion of Cr, and therefore, metals cannot form a continuous protective Cr_2_O_3_ layer for a long time [11].

The thermal shock resistance of nickel-based alloys was conducted by simulating the oxidation behavior under the operation and parked state of gas turbines. Studies revealed that Superni76, Superni750, and In600 had an excellent resistance to cyclic oxidation since a protective oxide scale consisting of Cr_2_O_3_ could form at 750 to 950 °C [12,13]. However, some researchers believed that the NiCr_2_O_4_ oxide film, resulting from the solid-state reaction between NiO and Cr_2_O_3_, is easily peeled off from the substrate under the cyclic oxidation, because of the high growth stress at 900 °C [14]. Besides the long time oxidation and cyclic oxidation, Ni-based alloys used in gas turbines also face other corrosion, especially salt corrosion [15,16]. When gas turbines are used in marine environments and thermal power conditions, the hot corrosion behavior of the Ni-based superalloys in a high temperature environment containing salt is one important issue. It was found that Na_2_SO_4_ could provide SO_3_ and O^2−^ at high temperatures, which led to the formation of MS_x_ and ${\mathrm{MO}}_{x}^{y-}$, promoting cracking and peeling off of the oxide film, and finally accelerated corrosion [17,18]. On the other hand, Cl^−^ could react with metals and oxides, and form ${\mathrm{MO}}_{x}^{y-}$ and Cl_2_ at high temperatures. Cl_2_ could penetrate into the inside of the oxide film easily and further react with the oxides, leading to the peeling off of oxide films, and accelerating the corrosion of the alloy [19,20,21]. 

As previous studies usually focused on the corrosion behavior of superalloys in a single environment, a more intensive study concerning the environmental details and their relationships with the corrosion of aircraft engine blades is urgently required. In fact, engine blades are used in elevated temperatures and high salt environments in the marine environment during operation and parking, respectively. In this environment, the blade suffers synergistic corrosion between high oxidation and low temperature electrochemical reactions. When working in high temperatures, oxidation happens; on the other hand, when parked in normal temperatures, the electrochemical reaction happens. Therefore, the metal materials used in aircraft engine blades experience a special corrosion: An alternating high temperature oxidation and normal temperature electrochemical reaction. However, limited published papers have focused on these alternating oxidation and electrochemical reactions.

In this paper, the alternating corrosion behavior of GH4169 alloy (the blade material) under the alternating high-temperature oxidation (900 °C) and normal temperature corrosion (3.5% NaCl solution, 30 ± 1 °C) were investigated. The corrosion rate was calculated by weighing experiments, and the composition and morphology of the corrosion products were analyzed by XRD, XPS, and SEM. The alternating corrosion mechanism was discussed.

## 2. Materials and Methods 

### 2.1. Material and Specimens

The material used in this study was Ni-based superalloy GH4169, with a chemical composition as listed in Table 1. After cut into a piece with the dimensions of 10 mm × 10 mm × 2 mm, the specimens were ground using SiC papers up to 2000 grit, cleaned with distilled water and alcohol, and dried in cold air before the corrosion tests.

### 2.2. Alternating Oxidation and Normal Temperature Corrosion Test

**Figure 1:**
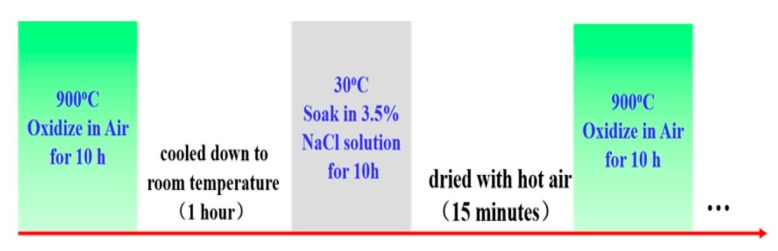
Schematic of the experimental cycles: alternating oxidation (at 900 °C) and solution emersion (in 3.5% NaCl solution, 30 ± 1 °C).

The specimens were suspended by a quartz and inserted inside a tube furnace, in which it was first performed in static air conditions at 900 °C for 10 h. After 10 h of oxidation, these specimens were cooled down in the air to room temperature for 1 h and immersed in a 3.5% NaCl solution at 30 ± 1 °C for another 10 h. Then, the specimens were dried with hot air for 15 min and NaCl was deposited evenly on the surface of the specimens. Next, the specimens were put into the tube furnace to begin the next experimental cycles. Each experimental cycle was 20 h, including 10 h of oxidation and 10 h of normal immersion in 3.5% NaCl solution. The whole test included 10 cycles, as shown in Figure 1. Specimens after high temperature corrosion were recorded as No. x. 5 cycle (x is the number of cycles, x = 0, 1, 2, … 10, the same below), and those after normal temperature corrosion were recorded as No. x cycle.

**Figure 2:**
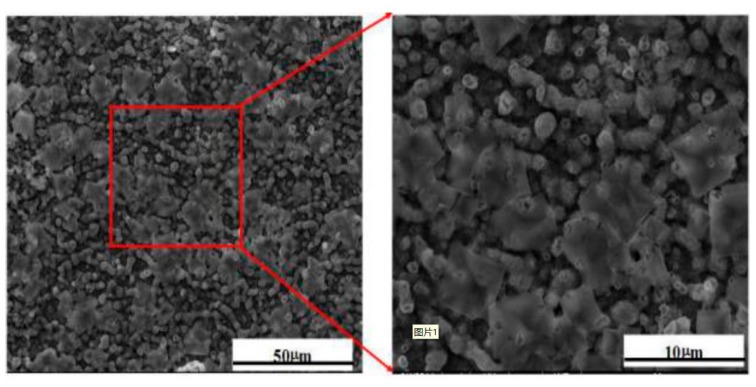
Surface morphology (SEM, SEI) of the NaCl deposit on the surface of the oxide scale after No. 0.5 and 1 cycles (oxidation at 900 °C and emersion in 3.5% NaCl solution).

After each half cycle, all the samples were weighed using an electronic balance with an accuracy of 0.01 mg. There were five parallel samples in each weighing experiment. To ensure reasonable reproducibility, all the weight gain tests were normally repeated at least four times for each specimen. Each experiment was repeated at least three times. The surface morphology of the NaCl deposit is shown in Figure 2, which shows that the NaCl layer is uniform over the surface of the specimen. The average weight of the NaCl deposit was about 0.1mg/cm^2^.

### 2.3. Experimental Methods

The surface and cross-section morphologies of the oxides were observed by a field emission scanning electron microscope (SEM) (FEI INSPEWCT F50, Hillsboro, OR, USA) equipped with an energy dispersive spectrometer (EDS) (INCA, X-Max, Oxford instruments Co., Oxford, UK). The acceleration voltage of the applied scanning electron microscope is 25 kV, both of which used secondary electron images (SEI) and backscattered electron images (BEI).

The distribution of the elemental elements of the oxide film was detected by an electron probe micro analyzer (EPMA). The EPMA equipment was a Shimadzu Model EPMA-1610 electron probe micro analyzer (Kyoto, Japan) at a 15 kV accelerated voltage. 

X-ray diffraction (XRD, X’pert PRO, Panalytical, Almelo, The Netherlands) was used to determine the phase composition of the oxide film. A step-scanning X-ray diffractometer was utilized, with Cu Kα radiation, in the scanning range of 10 to 90°.

The valence composition of the chemical elements in the corrosion products was examined by an ESCALAB 250 X-ray photoelectron spectroscopy (XPS, Waltham, MA, USA). Al Kα1486.6 eV was chosen as the monochromatic X-ray source. The light was 500 μm in diameter, and the pass energy was 50 eV. The binding energy of the carbon adsorption energy calibration was 284.6 eV.

## 3. Results

### 3.1. Kinetics of Hot Corrosion

**Figure 3:**
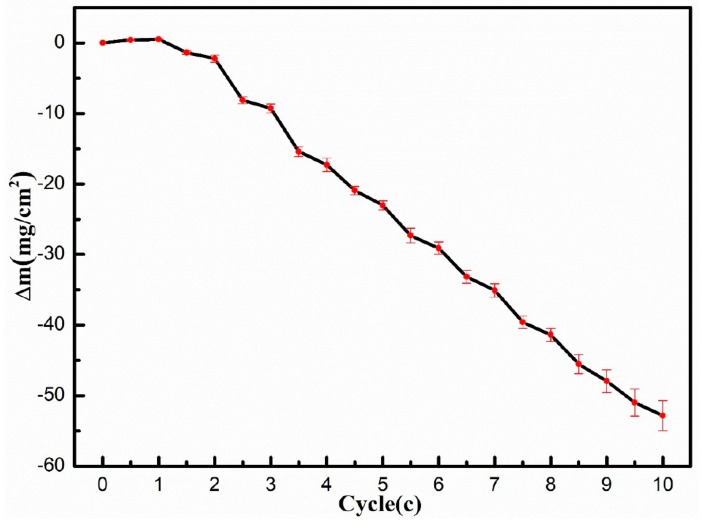
Weighing curve of GH4169 in 10 cycles of a high and low temperature alternating environment (oxidation at 900 °C and emersion in 3.5% NaCl solution).

Figure 3 shows the weight gain curve of the sample under alternating corrosion tests. The results show that the mass gain was only obtained after the first cycle, including the 0.5 cycle and 1cycle. After the other cycles, the weight of the specimens decreased, thus the weight loss was obtained for the remaining experimental cycles. Additionally, the weight loss increased with the increasing cycle number. The weight loss increased up to 52.8 ± 2.1 mg/cm^2^ of the No.10 cycle. In addition, weight loss occurred in both the oxidation at 900 °C and normal immersion at 30 °C from the No.1.5 cycle to the No.10 cycle.

### 3.2. XRD Analyses

**Figure 4:**
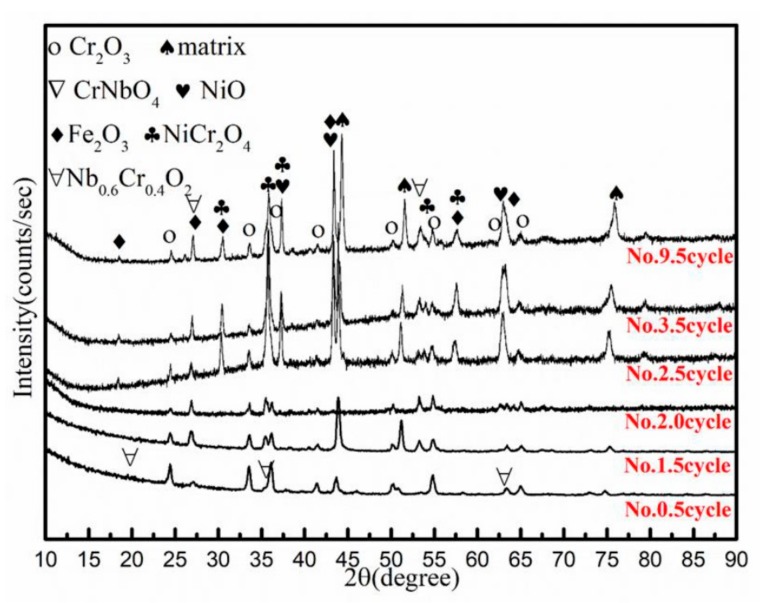
XRD results of the corrosion products remaining on the sample surface (No. 0.5, 1.5, 2.5, 3.5, 9.5 cycle) and spalling corrosion product powder filtered from 3.5%NaCl solution after No. 2 cycle.

The compositions of the corrosion products after different cycles were analyzed by XRD. The XRD analysis revealed the corrosion products and matrix information of samples after different cycles, as shown in Figure 4. The result shows that, except the matrix information, the corrosion products consisted of Cr_2_O_3_ and CrNbO_4_ after the No. 0.5 cycle. It indicates that an oxide layer mainly composed of Cr_2_O_3_ formed on the surface of the material after the 900 °C oxidation. From the XRD results, CrNbO_4_ in the oxide film was converted to Nb_0.6_Cr_0.4_O_2_ after the No. 1.5 cycle. In fact, after the 1.5 cycle, the corrosion products were not compact and loose because we also measured the corrosion product powders filtered from the 3.5% NaCl solution after two cycles, as shown in Figure 4. We noticed that the corrosion products on the samples were mainly Cr_2_O_3_ and Cr-Nb oxides after two cycles. Additionally, some powders remained in the immersion solution. We filtered them and tested them. We found that the remaining powders were the corrosion products formed on the samples. They were still Cr_2_O_3_ and Cr-Nb oxides. This result indicates that after the 1.5 cycle, although Fe and Ni oxides did not form, the corrosion products filled with Cr_2_O_3_ and Cr-Nb oxides are not compact, like that formed after the 0.5 cycle. Corrosion products can peel off in the immersion solution. In the XRD of the corrosion product powders, there is no matrix information. When the specimen was oxidized after the 2.5 cycle, new oxides, such as NiO, Fe_2_O_3_, and NiCr_2_O_4_, were detected by XRD analysis. From the No. 2.5 cycle to the No. 9.5 cycle, the phase composition of the corrosion products’ film changed slightly while a weakened intensity of the peak of Cr_2_O_3_ was detected, suggesting a decrease of the Cr_2_O_3_ content. The XRD results show the change of the corrosion products. From the initial main Cr_2_O_3_ layer, the corrosion products changed to be NiO, Fe_2_O_3_, and NiCr_2_O_4_.

### 3.3. Morphologies of the Oxides

**Figure 5:**
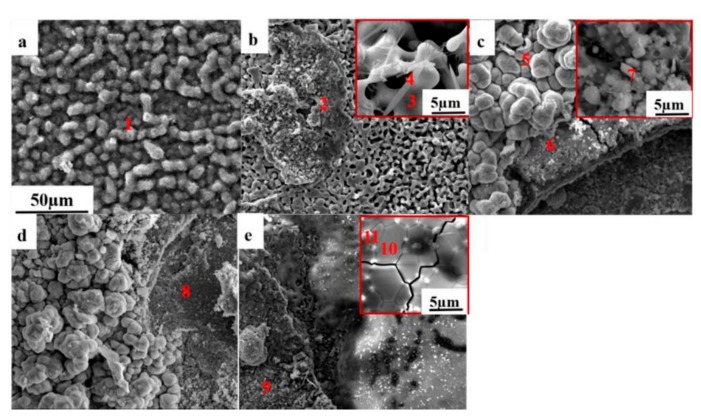
SEM surface morphologies (SEM, SEI) of corrosion products formed after (**a**) No. 0.5 cycle, (**b**) No. 1.5 cycle, (**c**) No. 2.5 cycle, (**d**) No. 3.5 cycle, and (**e**) No. 9.5 cycle.

The SEM surface morphologies of the corrosion oxides at different cycles are shown in Figure 5a–e. The EDS analysis of the oxides is shown in Table 2. At the No. 0.5 cycle, a dense and convex Cr_2_O_3_ film formed on the surface of the specimen (point 1). After the No. 1.5 cycle, the oxide film formed lots of defects, which became loose and began spalling, with many holes on the spalling surface. Combined with the EDS and XRD analysis, the oxides in the spalling areas were mainly composed of NiO and Fe_2_O_3_ with a small amount of Al_2_O_3_, while the oxide of unpeeled areas was Cr_2_O_3_ (point 2–4). The morphologies of the oxide films changed to more complicated triple layers after the No.2.5 cycle. The oxides in the outer layer consisted of NiO and Fe_2_O_3_. The intermediate layer, which had crackle, was mainly NiO, while the white granular oxide was NiO mixed with a small amount of Fe_2_O_3_ (point 6). The inner layer with a small number of pores consisted of NiO and Fe_2_O_3_, with lots of Cr_2_O_3_ beside the pores (point 7). After the No.3.5 cycle, the morphologies of the oxide film did not change significantly compared with the No. 2.5 cycle, and the compositions of the oxide scales at point 5 and point 8 were similar. After a severe spalling, the oxide scales formed at the No.9.5 cycle were much denser than those formed before. The outer oxide layer was composed of NiO and Fe_2_O_3_ (point 9). On the inner oxide scale, the grains grew up gradually as the cycles increased, and cracks along the grain boundary expanded. It consisted of NiO (point 10), with a small amount of Nb-rich oxides beside the grain boundaries (point 11). From the surface morphologies of the oxides’ film, we can see that the protective Cr_2_O_3_ layer formed after the 0.5 cycle disappeared after immersion in 3.5 NaCl solution. The GH4169 alloy suffered serious corrosion after the 1.5 cycle, and lots of corrosion oxides, including NiO and Fe_2_O_3_, formed on the specimens.

**Figure 6:**
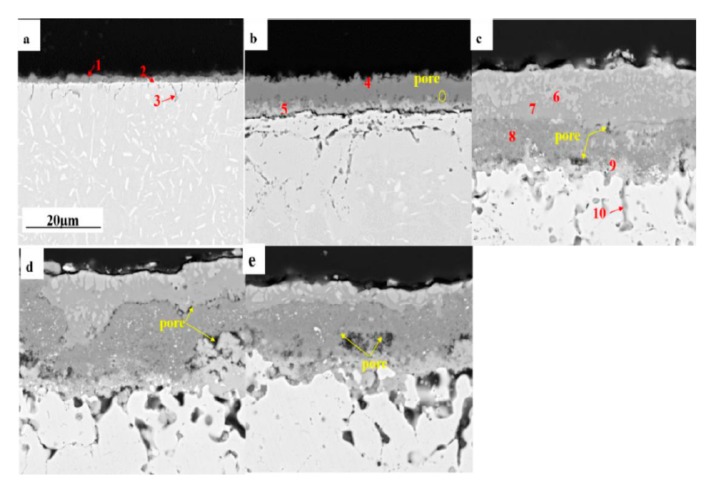
SEM cross-section morphologies (SEM, BEI) of corrosion products formed after (**a**) No. 0.5 cycle, (**b**) No. 1.5 cycle, (**c**) No. 2.5 cycle, (**d**) No. 3.5 cycle, and (**e**) No. 9.5 cycle.

Figure 6a–e shows the cross-section morphologies of the corrosion oxides at different cycles. The corresponding EDS analysis of the oxides is shown in Table 3. After the No. 0.5 cycle, a dense Cr_2_O_3_ formed on the surface (point 1). The internal oxidation of Al-rich oxides was detected as shown in Table 3 (point 3). A dense oxide formed at the interface between the outer oxide layer and the substrate, consisting of CrNbO_4_ and a small amount of Ti and Nb oxides (point 2). After the No. 1.5 cycle, the oxides film had a multi layered structure. The outer layer with a few small holes was thicker than the No. 0.5 cycle, which mainly consisted of Cr_2_O_3_ (point 4). Spalling observations were made at the interface between oxides and based metals. Additionally, above the based metal, the inner layer with some cracks consisted of Cr, Nb, and Ti (point 5). The internal oxidation was more serious than the 0.5 cycle. After the No. 2.5 cycle, a thicker oxide scale formed with three layers, which shows that serous corrosion to the alloy at that time. A dense outmost layer consisting of NiO and Fe_2_O_3_, an intermediate layer with many large pores consisting of Cr_2_O_3_, and an innermost layer of Nb_0.6_Cr_0.4_O_2_ and Nb and Ti -oxides (point 6–9) were formed. Meanwhile, internal oxidation was much more serious than before. From the No. 3.5 cycle to the No. 9.5 cycle, the oxide scales also consisted of three layers, and the phase composition changed slightly. However, as the test cycle increased, the number and size of the pores in the oxide scale increased. The thickness of the outset layer reduced, because of the spalling of the oxide scale. Actually, the internal oxidation was more and more serious.

**Figure 7:**
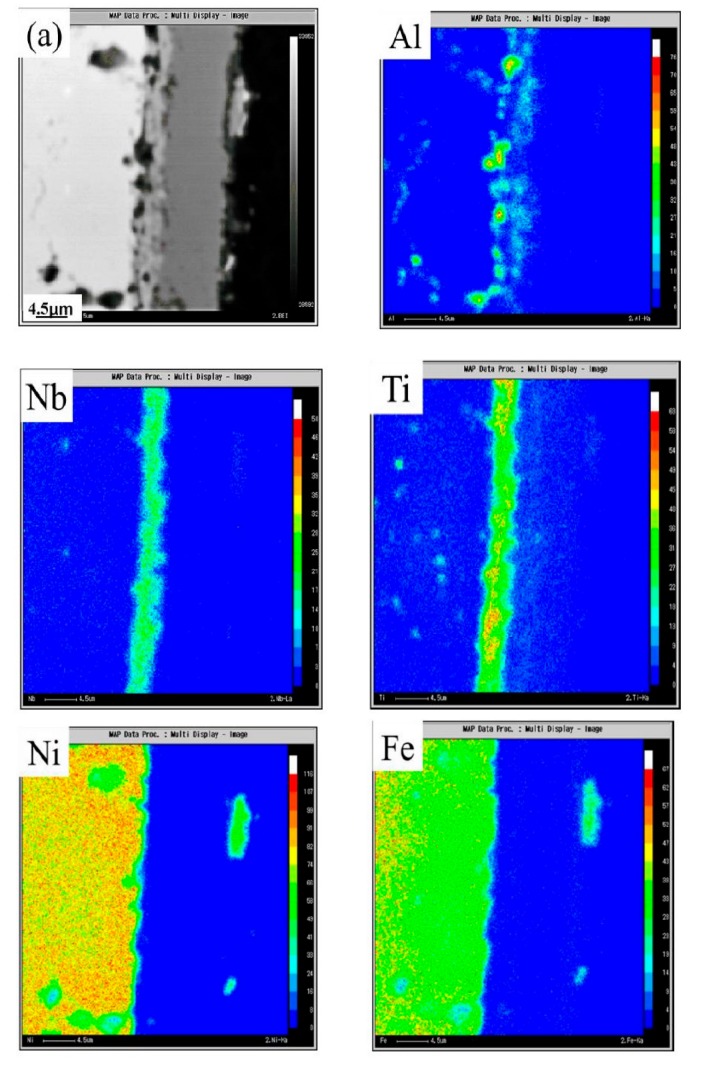

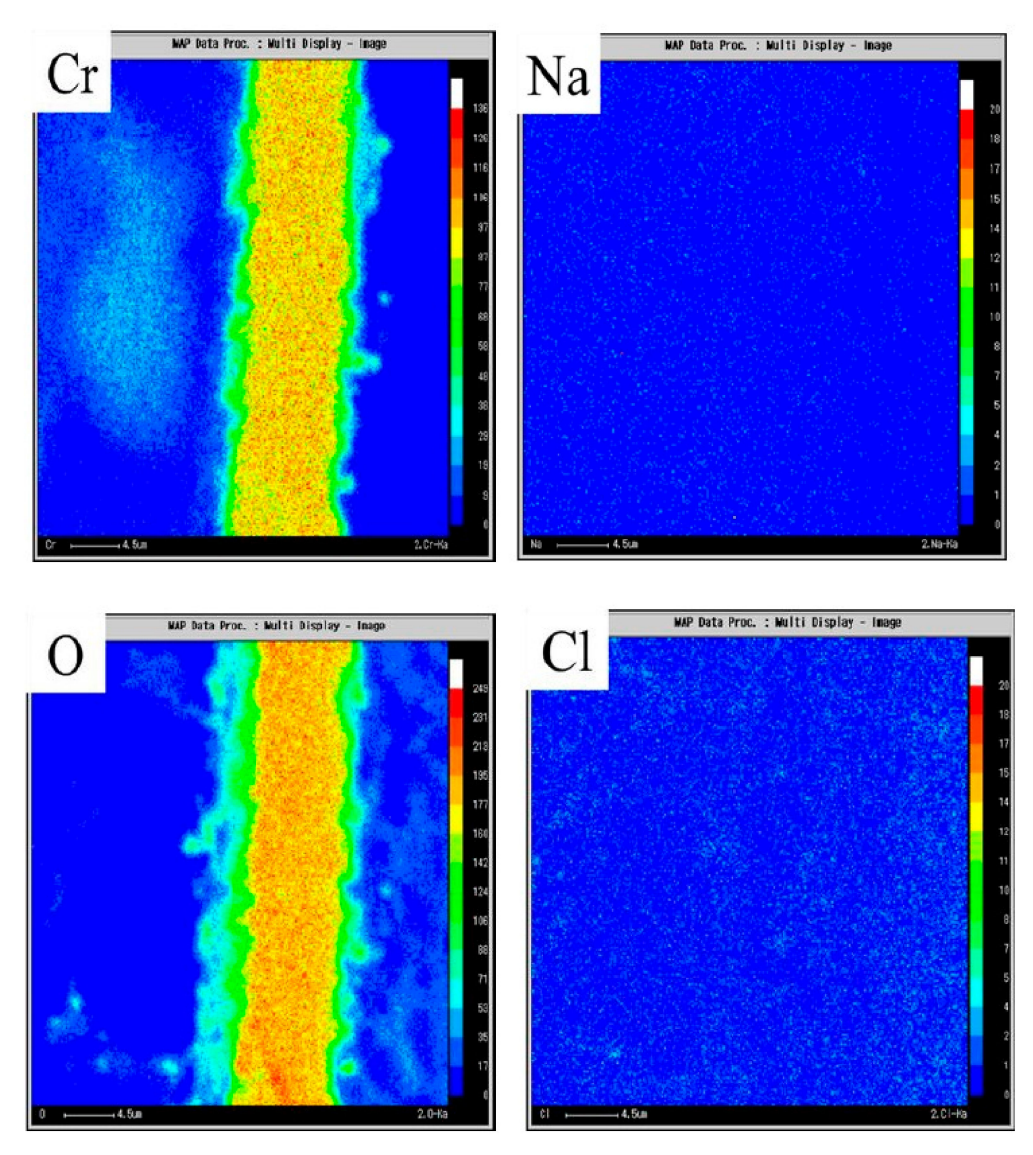

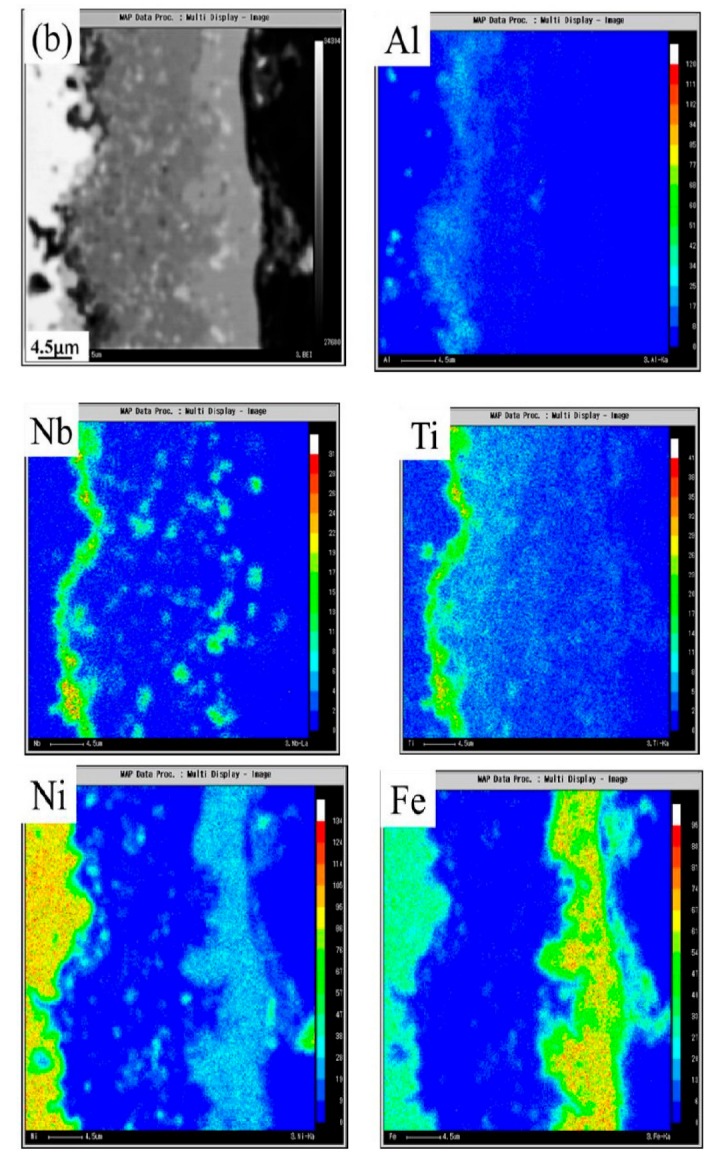

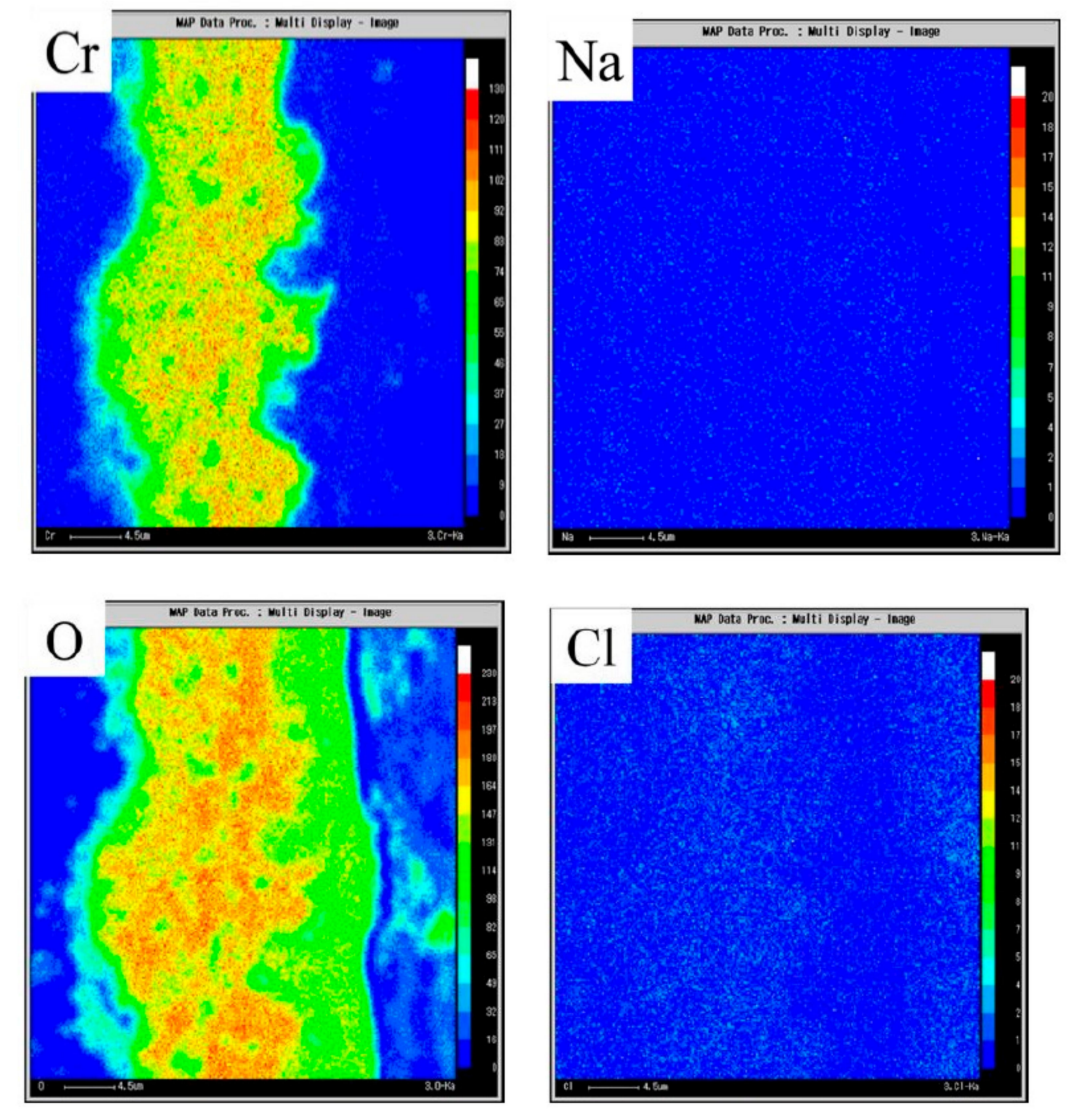
EPMA images of corrosion products after (**a**) No. 1.5 cycle and (**b**) No. 2.5 cycle.

The above experimental data show that the structures of oxide scales in the No. 1.5 cycle and No. 2.5 cycle changed more significantly. To better characterize the oxide scale formed on the superalloy GH4169 at this period, EMPA analyses were conducted. The elemental distributions in the oxide scale for the GH4169 samples oxidized under different cycles are shown in Figure 7. At the No.1.5 cycle, the outer layer was mainly composed of Cr-rich oxides. Beneath this layer, a mixed oxide layer with Nb and Ti-rich exists. The internal oxide was Al-rich oxide. Besides, Ni and Fe did not form a stable oxide. After 2.5 service cycles, the distributions of the corrosion product elements changed significantly. The outmost layer was composed mainly of Ni and Fe. The intermediate layer was composed of Cr. The innermost layer was rich in Nb and Ti. However, Ti and Nb oxides also diffuse into the outer layer. This transformation indicates that the corrosion products destroy the protection of Cr_2_O_3_. Large amounts of Ni and Fe oxides form in the outmost layer. This multi-oxide mixing can result in poor matching of oxide layers, thus causing the oxide layers to crack and spall. Therefore, the massive oxides spalled off from samples and weight loss became more and more serious.

**Figure 8:**
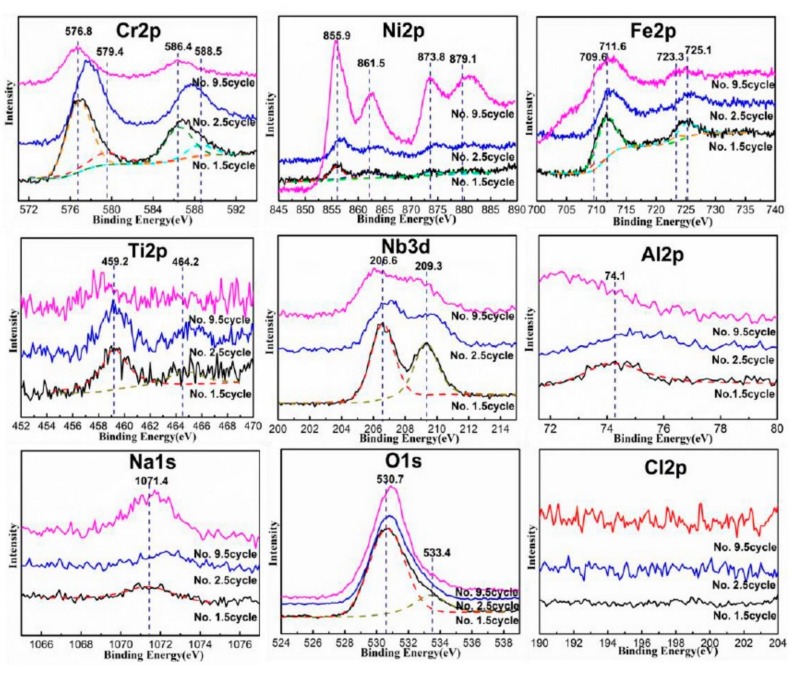
The XPS spectra of Cr 2p, Ni 2p, Fe 2p, Ti 2p, Nb 3d, Al 2p, Na 1s, O 1s, and Cl 2p of corrosion products formed after the No. 1.5 cycle, No. 2.5 cycle, and No. 9.5 cycle.

Some compounds were not detected in XRD analysis, possibly due to the volume fractions of those compounds are less than 5% [22]. To better characterize the corrosion product, XPS analyses were conducted. The spectra of Cr2p, Ni2p, Fe2p, Ti2p, Nb3d, Al2p, Na1s, Cl2p and O1s acquired by XPS on the oxides film formed at different cycles are shown in Figure 8. The Ni 2p spectrum exhibits Ni^2+^ peeks at 855.9 and 861.5 related to NiO and NiCr_2_O_4_ [23,24]. As the cycles increased, the Ni-oxides increased significantly, consistent with the XRD results. The Fe 2p spectrum exhibited a Fe^2+^ peek at 709.6 and a Fe^3+^ peek at 711.6 related to FeO and Fe_2_O_3_, respectively [25]. The spectra of Ti 2p, Nb3d, and Al2p corresponded to TiO_2_, Al_2_O_3_, Nb_2_O_5_, and CrNbO_4_, respectively. However, due to the low content of Ti, Nb, and Al in the superalloy, their peak signals were weak. Their phase could not be detected by XRD analyses. The spectra of O 1s results in two contributions originating from dissolved oxygen at 533.4 eV and oxide oxygen at 530.7 eV [26,27,28].

**Figure 9:**
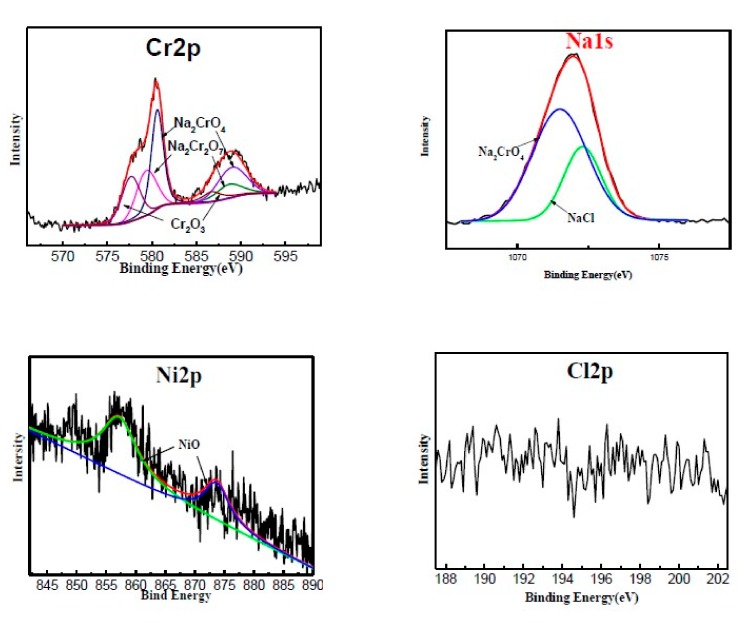
The XPS spectra of Cr 2p, Na 1s, Ni 2p, and Cl 2p of the collecting volatile corrosion products.

The above experimental results show that there is no NaCl or chlorides on the surface of samples after high temperature oxidation, which indicates that the chloride may be volatile. Therefore, volatile corrosion products piled on the quartz silks and tube inner wall above the furnace were collected from the suspended quartz silks and tube over the muffle furnace and analyzed by XPS. The spectra of Cr 2p, Fe 2p, Cl 2p, and Na 1s acquired by XPS on the collected volatile corrosion products are shown in Figure 9. The Cr2p spectrum exhibits that the products consisted of Na_2_Cr_2_O_7_, Cr_2_O_3_, and Na_2_Cr_2_O_4_. Meanwhile, the spectra of Na1s also shows the existence of Na_2_Cr_2_O_7_ and Na_2_Cr_2_O_4_. However, Cr_2_O_3_ and Na_2_Cr_2_O_4_ cannot be volatilized at 900 °C, and they should be produced by decomposition of Na_2_Cr_2_O_7_ [29]. The spectra of Ni2p indicates that the corrosion products contain NiO [24]. The spectra of Cl2p indicates that there is no Cl in the volatile corrosion products. Chlorides combined with oxygen form M_x_O_y_ and Cl_2_, which leads to chlorides not being detected [22,30].

The spectrum of Cr2p not only shows the existence of Cr_2_O_3_ and NiCr_2_O_4_ (576.8 and 586.4), but also characterizes the corrosion product of Cr^6+^ [31]. Considering the spectra of Na 1s, the corrosion products of Cr^6+^ may be Na_2_Cr_2_O_7_ and Na_2_Cr_2_O_4_. However, Na_2_Cr_2_O_7_ is a highly volatile and decomposed compound at 900 °C, and Cr^6+^ in the oxide is considered to be Na_2_Cr_2_O_4_. The spectrum of Cl2p indicates that there is no Cl compound in the corrosion product at all.

## 4. Discussion

### 4.1. The Mechanism of NaCl on Corrosion

The results obtained from this study clearly show that this alternating corrosion, including oxidation and solution immersion, increased the corrosion rate of GH4169. The obvious corrosion happened after immersion and during oxidation. After first oxidation at 900 °C, GH4169 formed a protective Cr_2_O_3_ layer, but it was destroyed after 1.5 cycle oxidation. The main reason should be that NaCl deposited on the surface of the sample reacted with Cr_2_O_3_ at 900 °C. The reactions of NaCl (s) with Cr_2_O_3_ are presented in Equations (1) and (2), in which the products of Na_2_CrO_4_, Cl_2_ and Na_2_Cr_2_O_7_ were formed. If NaCl is in the solid phase, the Gibbs free energy has a positive value. The same results were obtained in [32,33]. Although the theoretic calculation results show it is impossible for the reaction to happen, the reactions also are controlled by the products of Cl_2_ and the partial pressure of Cl_2_ [3]. If the partial pressure of Cl_2_ is low enough, the reactions can happen [3]. In this work, the partial pressure of Cl_2_ produced at equilibrium by Equation (1) at 900 °C is 2.99 × 10^−5^ atm, which was calculated by software HSC chemistry 6 (produced by OUTOKUMPU). This value of Equation (2) is 2.25 × 10^−15^ atm at the same temperature. Considering the Cl_2_ in this work is produced in the oxide layer, the partial pressure should be low, which may be lower than the critical partial pressure of Cl_2_. Combined with the experimental results, we think reaction (Equations (1) and (2)) can happen. However, Na_2_Cr_2_O_7_ is unstable and prone to decomposition through the reaction of Equation (3) at 900 °C, which finally forms Na_2_CrO_4_ and Cr_2_O_3_ [33]. The Gibbs free energy of Equation (3) was calculated to be a negative value by software HSC chemistry 6, which means this reaction can happen easily. The XRD results testified this analysis. Therefore, NaCl destroyed the protective Cr_2_O_3_ layer.

2NaCl (s) + 1/2Cr_2_O_3_ (s) + 5/4O_2_ (g) = Na_2_CrO_4_ (s) + Cl_2_ (g), ΔG_900_ °_C_ = 91.5 Kcal/mol (1)

2NaCl(s) + Cr_2_O_3_(s) + 2O_2_(g) = Na_2_Cr_2_O_7_(s) + Cl_2_(g), ΔG_900_ °_C_ = 78.6 Kcal/mol (2)

Na_2_Cr_2_O_7_(s) = Na_2_CrO_4_(s) + 1/2 Cr_2_O_3_(s) + 3/4O_2_ (g), ΔG_900_ °_C_ = −56.8 Kcal/mol (3)

Meanwhile, Cl_2_ and Na_2_Cr_2_O_7_ have volatile properties, and the volatilization of them can lead to defects, such as holes, to form in the oxide scales. Defects can reduce the fracture toughness of scales, and it is difficult to release the stress in a thermal shock environment. After two cycles, we found that the oxides peeled off from the samples, which testified this. Moreover, Cl_2_ (g) may penetrate into the substrate through the pores and cracks in the scale, and may reach the metal/scale interface [33,34]. Once the partial pressure of Cl_2_ (g) increases at the interface between the metal and scale, the metal elements are unstable and can easily form the reaction by Equations (4) and (5) [35,36]: 

Cr (s) + 3/2Cl_2_ (g) = CrCl_3_(g), ΔG_900_ °_C_ = −76.6 Kcal/mol (4)

Ni (s) + Cl_2_ (g) = NiCl_2_(g), ΔG_900_ °_C_ = −28.6Kcal/mol (5)

Chlorides (CrCl_3_ and NiCl_2_) may escape from the substrate/scale interface, and then the oxygen partial pressure is increased and the easy reaction of Equations (6) and (7) can occur [29,30]. The Gibbs free energies of Equations (4)–(7) were also negative, both of which mean reactions can happen. The volatilization of MCl_x_ causes more defects to form in the oxide scale, resulting in its spalling beingserious [33]. Therefore, the Cr_2_O_3_ layer was destroyed and lots of pores appeared under 900 °C at 1.5 cycles. The immersion in NaCl solution produced NaCl deposits on the surface of samples, which can be seen in Figure 2. NaCl reacted with oxides, which destroyed Cr_2_O_3_ and also promoted the formation of defects in the oxide scale. Subsequently, the sample was immersed in 3.5%NaCl aqueous solution, resulting in the dissolution of Na_2_Cr_2_O_4_ in the external and internal scales. Moreover, salt water entered the oxide layer to destroy the compactness of oxides. Meanwhile, the outward evaporation of H_2_O caused the rapid dehydration of oxide layers and decreased the compactness of oxide layers, too. The residual NaCl reacted again with the oxides to destroy the oxide layer more rapidly at high temperatures. Repeated alternation results in the disappearance of protective oxides, thus accelerating the corrosion of GH4169 alloy. 

2CrCl_3_ (g) + 3/2O_2_ (g) = Cr_2_O_3_ (s) + 3Cl_2_ (g), ΔG_900_ °_C_ = −48.3 Kcal/mol (6)

NiCl_2_ (s) + 1/2O_2_ (g) = NiO (s) + Cl_2_ (g), ΔG_900_ °_C_ = −0.5 Kcal/mol (7)

### 4.2. Corrosion Mechanism 

**Figure 10:**
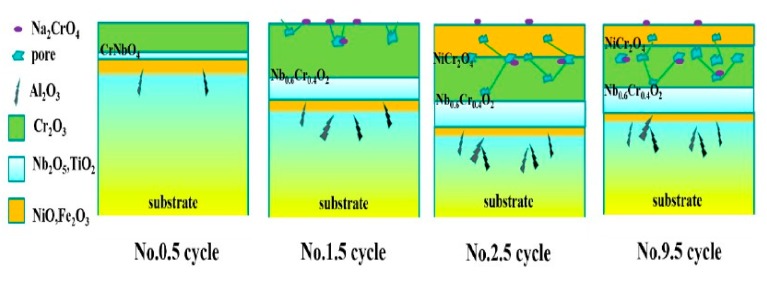
Schematic of GH4169 during the corrosion process under alternating oxidation at 900 °C and solution emersion.

Schemes of the corrosion mechanism of GH4169 during the alternating high-temperature oxidation (900 °C) and normal temperature corrosion (3.5% NaCl solution, 30 ± 1 °C) are illustrated in Figure 10. A dense and protective Cr_2_O_3_ film was formed on the surface due to selective oxidation of Cr elements at the No. 0.5 cycle, as shown in Figure 5. Interface oxides of Nb_2_O_5_ can occur during phase reaction with Cr_2_O_3_, as shown in Equation (8), to form CrNbO_4_, shown in the 0.5 cycle picture. After the immersion in 3.5% NaCl, a large amount of NaCl deposited on the oxide layer, as shown in Figure 2. Then, at the next oxidation, deposited NaCl reacted with the oxides, which destroyed the compact Cr_2_O_3_ and produced defects in the oxide scale as shown in the 1.5 cycle picture. The defects also promoted the spalling of the outer layer of Cr_2_O_3_ and accelerated the depletion of Cr. The corrosion products peeled off in the immersion solution, which was testified by the XRD result. The destruction of the continuous protective Cr_2_O_3_ scale produced the formation of Ni\Fe oxides. The defects provided channels for the diffusion of O_2_, which promotes the oxidation of Nb at the substrate/scale interface. Ni was oxidized to NiO, and then reacted with Cr_2_O_3_ to form a compact spinel structure of the NiCr_2_O_4_, which could prevent more efficiently chlorination and oxidation. The interaction between them causes the reduction of Cr:Nb in interface oxides, as shown in Equation (8) [37,38]. There is not enough Cr_2_O_3_ to react with Nb_2_O_5_, thus CrNbO_4_ transformed into Nb_0.6_Cr_0.4_O_2_ [39].

Nb_2_O_5_ + Cr_2_O_3_ = 2CrNbO_4_ (8)

A mixed outermost layer of NiO and Fe_2_O_3_ and an intermediate Cr_2_O_3_ layer were formed after the No. 2.5 cycle, as shown in Figure 7. The continuous Cr_2_O_3_ layer was destroyed, and then NiO and Fe_2_O_3_ formed. These mixed oxides piled on the surface of samples, so the oxide scale shows a more compact morphology than the No. 1.5 cycle (in Figure 5). The residual oxide scale is not protective. Ni and Fe can diffuse the outside of the Cr_2_O_3_ scale. The chemical reaction between NaCl and Cr_2_O_3_ results in changes of the structure and composition from the No. 1.5 cycle to the No. 2.5 cycle. At the same time, NiO reacts with Cr_2_O_3_ to form NiCr_2_O_4_ in the solid phase of Equation (9) [40,41,42]:

NiO + Cr_2_O_3_ = NiCr_2_O_4_ (9)

From the No. 2.5 cycle to the No. 9.5 cycle, the outer oxide continuously peeled off, which was testified by SEM, XRD, and so on. The outward diffusion rate of the elements could not keep up with the spalling speed of the oxide scale, resulting in a decrease of the oxide scale thickness. Additionally, the inward diffusion of oxygen is increased by defects, and a large number of internal oxides are more and more serious under the outer scale [43,44]. The structure and composition of the outer layer of oxides scale changed significantly throughout the whole corrosion process. Otherwise, Nb with a higher valence than that of Ti is expected to decrease the concentration of oxygen vacancy and thus form a stable Nb and Ti-oxides interface layer [45].

## 5. Conclusions

GH4169 alloy suffered serious corrosion during the alternating at high-temperature oxidation and normal temperature corrosion. The deposited NaCl on the surface of samples after immersion reacted with Cr_2_O_3_ at the next oxidation, which is the main reason this serious corrosion was produced. The deposit, NaCl, reacted with Cr_2_O_3_ to form corrosion products, such as Cl_2_, Na_2_Cr_2_O_4_, Na_2_Cr_2_O_7_, and CrCl_3_, which consume Cr_2_O_3_ and promote the formation of defects inside the oxide scale. The outer oxide layer was changed from Cr_2_O_3_ to a mixed oxide of NiO and Fe_2_O_3_. After repeated iterations, the mixed oxides resulted in spalling of the oxide film because the fracture toughness of the corrosion scale was reduced.

## Figures and Tables

**Table 1:** The compositions of GH4169 alloy.

**Element**	C	Cr	Mo	Al	Ti	Fe	Nb + Ta	B	Ni
**Mass%**	0.045	19.09	3.25	0.88	0.83	18	5.08	0.05	Bal.

**Table 2:** The EDS results of points shown in Figure 5.

Element	Cr (at%)	Fe (at%)	Ni (at%)	Nb (at%)	Ti (at%)	Al (at%)	O (at%)
1	19.4	0.3	1.3	0.5	1.3	-	77.1
2	10.0	1.7	1.0	4.6	2.1	1.8	78.9
3	0.5	15.0	54.9	-	-	4.2	25.4
4	0.6	3.8	20.5	-	-	15.3	59.9
5	3.3	13.9	13.6	-	0.3	-	68.8
6	0.7	2.5	36.0	-	-	-	60.8
7	14.2	3.2	4.8	1.4	0.8	0.9	74.7
8	5.0	17.3	16.5	-	0.4	-	60.8
9	4.8	17.3	12.7	-	-	-	65.2
10	-	0.9	33.3	-	-	-	65.8
11	-	1.1	33.7	3.6	-	-	61.6

**Table 3:** The EDS results of points shown in Figure 6.

Element	Cr (at%)	Fe (at%)	Ni (at%)	Nb (at%)	Ti (at%)	Al (at%)	O (at%)
1	17.5	1.7	0.5	3.0	4.0	2.4	70.8
2	6.2	4.9	34.8	9.8	1.0	1.5	41.8
3	9.4	13.0	34.7	2.1	1.6	10.7	28.6
4	33.6	-	-	-	-	-	66.4
5	27.2	-	-	12.9	3.7	1.7	54.5
6	0.5	4.1	41.6	-	-	-	53.8
7	0.9	24.1	16.4	-	-	-	58.6
8	31.8	0.5	1.5	-	0.3	-	65.8
9	10.5	0.9	3.1	13.4	3.7	1.3	67.2
10	1.3	13.4	51.0	-	-	5.5	28.8
